# Redefining Obstructive Sleep Apnea: Multidimensional Phenotyping Beyond the Apnea–Hypopnea Index

**DOI:** 10.3390/pathophysiology33020024

**Published:** 2026-03-30

**Authors:** Harjinder Singh, Nida Qadir, Malti Bhamrah, William Rosales-Gonzalez, Paul Bhamrah, Naomi Ghildiyal, Brittany Monceaux, Cesar Liendo, Sheila Asghar, Jonathan Steven Alexander, Oleg Y. Chernyshev

**Affiliations:** 1 Academic Comprehensive Sleep Medicine Program, Department of Neurology, Louisiana State University Health Shreveport, 1501 Kings Highway, Shreveport, LA 71103, USAoleg.chernyshev@lsuhs.edu (O.Y.C.); 2 Sleep Medicine Section, Department of Medicine, Baylor College of Medicine, 1 Baylor Plaza, Houston, TX 77030, USA; 3 Department of Molecular and Cellular Physiology, Louisiana State University Health Shreveport, 1501 Kings Highway, Shreveport, LA 71103, USA

**Keywords:** Q2 analysis, obstructive sleep apnea, phenotypes, polysomnography, hypoxic burden, respiratory effort–related arousals, cardiovascular risk

## Abstract

**Background:** Obstructive sleep apnea (OSA) is a complex and diverse disorder affecting almost one billion individuals worldwide. Severity of untreated OSA, measured by the apnea–hypopnea index (AHI), is noted to be associated with an increased all-cause and cardiovascular mortality. Although widely used, AHI insufficiently captures disease variability as there is a poor correlation of symptoms with the AHI. There lies individual susceptibility to the effects of OSA and that parameter alone poorly predicts cardiovascular outcomes without considering intermittent hypoxia and the hemodynamic effects of OSA. Recognition of clinical, polysomnographic, and neurophysiological phenotypes offers an opportunity to refine diagnosis, prognosis, and management strategies. **Methods:** We conducted a narrative synthesis of the literature involving 70 articles, focusing on quantitative and qualitative (Q2) clinical traits, polysomnographic parameters, and mechanistic insights that enable subclassification of OSA beyond AHI. Evidence from large cohorts, animal models, and pathophysiological studies were reviewed. **Results:** Phenotyping based on a Q2 analysis of polysomnographic respiratory event predominance, event duration, positional and REM dependence, hypoxic burden, and arousal characteristics reveals significant heterogeneity in risk profiles and therapeutic response. Apnea-predominant OSA correlates with a higher oxygen desaturation index and Epworth sleepiness scale. Hypopnea-predominant OSA correlates with a cardiometabolic disease burden and may show a more favorable response to surgical therapies. The duration of respiratory events is related to cardiovascular risk, and REM-predominant OSA independently predicts hypertension and adverse cardiovascular outcomes. Supine-predominant OSA demonstrates treatment responsiveness to auto-positive airway pressure and positional therapy. Respiratory effort–related arousals (RERAs), RERA-predominant OSA and the broader respiratory disturbance index (RDI) provide neurophysiological insight often missed by AHI-based classifications. Hypoxic burden, rather than AHI, emerged as a superior predictor of cardiovascular events and mortality. Finally, arousal frequency and periodic limb movements independently predict cardiovascular morbidity. **Conclusions:** Employing Q2-based phenotyping that incorporates clinical, polysomnographic, and neurophysiological markers improves risk stratification, prognosis, and individualized management of OSA. Future investigations should prioritize integrating phenotypic subclassification into diagnostic criteria and treatment planning to advance precision medicine in sleep apnea care.

## 1. Introduction

Obstructive sleep apnea (OSA) is a prevalent disorder marked by repeated episodes of upper airway collapse during sleep, leading to intermittent hypoxemia, hypercapnia, arousals, and sleep fragmentation [1]. It is known to affect almost one billion people globally, representing a major public health burden [2]. Traditionally, the apnea–hypopnea index (AHI), which counts the number of apneas and hypopneas per hour of sleep, remains the cornerstone for OSA diagnosis, severity grading, and treatment eligibility [3]. High AHI values have been linked to greater risks of cardiovascular, metabolic, and neurocognitive outcomes, such as hypertension, coronary artery disease, stroke, heart failure, arrhythmias, diabetes, and overall impaired quality of life [4]. Data from the Wisconsin Sleep Cohort indicate that individuals with an AHI above 30 events per hour face a higher risk of both overall and cardiovascular mortality [5]. Similarly, a cross-sectional analysis shows that those with an AHI of 20 per hour or more are more likely to experience stroke, compared to those with no sleep disordered breathing (AHI less than 5) [6]. Animal model studies also demonstrate that OSA with an AHI of greater than or equal to 20 leads to a worsening of ischemia/reperfusion mediated injury in the brain [7]. Retrospective analyses suggest that using positive airway pressure (PAP) therapy is linked to reduced all-cause mortality and a lower risk of major adverse cardiovascular events [8] and strokes [9]. Furthermore, a meta-analysis of both randomized controlled trials and confounder-adjusted non-randomized controlled studies reveal a benefit of PAP therapy in lowering all-cause and cardiovascular mortality in patients with OSA [10]. However, some randomized control trials have not found a clear association between CPAP use and secondary prevention of cardiovascular disease [11,12,13], possibly due to challenges such as the need for control groups and reliance on patient adherence.

In these studies, AHI is often the sole metric applied for diagnosing, prognosticating, and monitoring treatment response in OSA, a heterogenous disorder with considerable clinical and pathophysiological diversity [14]. This has sparked discussion about whether certain patient subtypes might derive greater cardiovascular benefit from treatment, despite having a similar AHI. Polysomnography provides a wealth of physiologic data, which provides the opportunity to identify various OSA phenotypes. In this context, phenotype is considered “A group of OSA patients differentiated from others by one or more disease characteristics that relate to clinically meaningful attributes, such as symptoms, response to therapy, health outcomes, and quality of life” [15]. A dialogue has been started to evaluate the severity of OSA beyond the AHI [16], and this review article aims to tabulate those parameters for a more mechanistic review to be used by different clinicians while interpreting different sleep studies.

Endotyping categorizes OSA based on its underlying pathophysiological or biological causes such as structural airway problems, decreased muscle response in the throat, heightened respiratory control system sensitivity (loop gain), and a lowered threshold for awakening during sleep [17]. The resulting OSA phenotypes reflect a combination of these mechanisms with genetic predispositions. Recognizing these phenotypes can help link specific patient types to expected clinical outcomes and may open doors to alternative therapies for those who cannot use PAP devices. These non-PAP alternatives include supplemental oxygen [18] and acetazolamide [19] to reduce loop gain, and medications such as hypnotics to decrease arousal threshold. For example, identifying patients with a specific type of palatal collapse (anteroposterior rather than concentric) can guide eligibility for hypoglossal nerve stimulators [20].

This review aims to outline the different endotypes and phenotypes of obstructive sleep apnea, highlighting their clinical relevance and providing a concise point-of-care guide for interpreting sleep studies. The significance of endophenotyping and recommended approaches for implementation have been discussed in previous multidisciplinary research efforts [21].

## 2. Materials and Methods

This work is a narrative review of published studies addressing quantitative and qualitative (Q2) analyses of OSA phenotyping. A literature search using PubMed and Scopus databases was conducted throughout January 2026 with the search terms ‘obstructive sleep apnea’, ‘phenotypes’, ‘hypoxic burden’, ‘obstructive apnea predominant OSA’, ‘hypopnea predominant OSA’, ‘RERA-predominant OSA’, ‘upper airway resistance syndrome (UARS)’, ‘REM-predominant OSA’, ‘respiratory effort related arousals’, ‘supine-predominant OSA’, and ‘sleep fragmentation’. We prioritized randomized controlled trials, large cohort studies, mechanistic investigations, translational animal research, and expert consensus statements. The studies were examined individually and filtered to ensure the ones best suited for this review were included. Articles were included if they addressed the classification of OSA beyond AHI based on qualitative and quantitative PSG analysis, explored neurophysiological traits, or evaluated cardiovascular, cerebrovascular, and neurocognitive consequences linked to phenotypic traits. A table summarizing the current evidence of various phenotypes with research gaps was generated (Table 1). This review is not a systematic review, but integrates evidence into thematic domains to provide a clinically meaningful synthesis.

## 3. Results

### 3.1. Phenotyping by Respiratory Event Predominance

Previous studies have discussed further classifying OSA based on the predominance of apnea or hypopnea events [22]. OSA pathophysiological endotypes include (1) high critical closing pressure (Pcrit), (2) inadequate responsiveness of the upper airway dilator muscles during sleep, (3) low respiratory arousal threshold, and (4) high loop gain of the respiratory control system [23]. Apneas occur due to static obstruction in the absence of flow and are dependent on the Pcrit or anatomical pathogenesis. Hypopneas represent flow limitation due to dynamic-obstruction-associated non-anatomical pathogenesis [24]. Demographic variations exist as patients with severe obesity (body mass index greater than 45) have been shown to have a very high hypopnea to apnea ratio with an abundance of hypopneas [25], which could be due to the different pathophysiological mechanisms of static and dynamic obstruction in the extremely obese.

A retrospective analysis that divided OSA patients into apnea-predominant, hypopnea-predominant, and RERA-predominant OSA (UARS) groups found that those with apnea-predominant OSA had higher oxygen desaturation index values and greater daytime sleepiness, as measured by the Epworth sleepiness scale, than the other groups (Table 2). The hypopnea-predominant group tended to be older, included more women, and had higher rates of hyperlipidemia, heart failure, and coronary artery disease compared to the apnea-predominant group. Both apnea and hypopnea-predominant patients had more hypertension and diabetes than those with RERA-predominant OSA. Importantly, after adjusting for confounders such as age, sex, and smoking history, a higher hypopnea index was associated with increased risks for coronary artery disease and heart failure [26].

However, some investigations have not found significant differences in risk between apneas and hypopneas alone [27], indicating that further research is needed in this area.

These classifications may also inform treatment selection. For instance, maxillomandibular advancement (MMA) surgery has shown higher success and cure rate in the hypopnea-predominant OSA compared to those with apnea-predominant OSA, with notable improvements in AHI, apnea index (AI), hypopnea index (HI), and oxygen desaturation index (ODI) metrics. This supports incorporating OSA phenotypes into clinical decision-making to personalize therapy [28].

### 3.2. Phenotyping by Respiratory Event Duration

The duration of respiratory events in OSA, and the impact of OSA on cardiovascular disease has been a focus of research. There has been a noted increase in the apnea event-related change in blood pressure and change in cerebral blood flow velocity (CBFV) with a longer duration of the event. Specifically, as apnea episodes lengthen from 10 s to more than 30 s, CBFV and blood pressure responses intensify markedly. This increase in mean apnea duration is also significantly associated with moderate-to-severe hypertension even after adjusting for age, sex, body mass index, current history of smoking, alcohol usage, AHI, and lowest oxygen desaturation [29]. Conversely, separate research indicates that shorter respiratory events are associated with higher all-cause mortality after adjusting for demographic factors, AHI, smoking, and prevalent cardiometabolic disease, which may be likely due to a greater extent of hemodynamic fluctuations caused by shorter respiratory events. The mean duration of respiratory events correlates more with hypopnea duration than apnea duration. Patients with the shortest events face substantially higher mortality risk, particularly in those with intermediate OSA severity [30]. Severe OSA is also characterized by shorter hypopneas and deeper average desaturations [31]. In addition, patients experiencing longer apneas (≥20 s) tend to have louder snoring, increased morning fatigue, and greater hypertension risk compared to those with shorter events (Table 2) [32].

Current evidence suggests that people experiencing shorter respiratory events in OSA may be more prone to unstable breathing patterns and heightened autonomic nervous system activity, which could elevate their risk for negative health outcomes. The arousal index, reflecting the body’s hemodynamic response following apneas and hypopneas, may partly explain why patients with both high AHI and brief respiratory events face an increased risk of OSA-related complications. These findings highlight the value of including event duration as part of OSA assessment and severity grading.

### 3.3. Phenotyping by Sleep Stage and Body Position

REM-predominant OSA, a phenotype more frequently observed in women, occurs when the REM AHI is disproportionately elevated relative to NREM AHI [33]. Ventilatory responses to chemostimulation are reduced during NREM sleep and are further reduced during REM sleep, which could possibly describe increased probability of having respiratory events [34]. Findings from the Wisconsin sleep cohort revealed that a REM AHI of 15 or more per hour is associated with hypertension, regardless of whether the overall AHI is below the diagnostic threshold [35]. This suggests that individuals who might not meet the standard OSA diagnostic criteria could still benefit from treatment if they have REM-predominant OSA, particularly those with uncontrolled hypertension. Severe OSA during REM is also associated with a greater risk of repeat cardiovascular events in patients with existing heart disease [36]. Supine-predominant OSA is seen in people with increased pharyngeal collapsibility, which markedly improves in the lateral position [37]. Clinical evidence shows that auto-adjusting PAP therapy, as opposed to fixed CPAP, can be more effective for these patients due to the variability in airway obstruction depending on sleep position (Table 2) [38]. Therefore, it is important to report both REM AHI and supine AHI in polysomnographic studies.

### 3.4. Phenotyping by Neurophysiological Traits

Respiratory effort-related arousals (RERAs) have been historically underrepresented in much of the earlier sleep research, despite their association with significant and potentially dangerous sleep apnea symptoms. RERAs are crucial markers to assess the neurophysiological burden of respiratory compromise and are independently associated with objective daytime sleepiness, beyond the effect of oxygen desaturating events [39]. RERAs should be calculated especially in the patients complaining of excessive daytime sleepiness, fatigue, insomnia, or any other neurocognitive symptoms. The American Academy of Sleep Medicine (AASM) scoring manual recommends including RERAs and considering PAP therapy if respiratory disturbance index (RDI) is more than 5 per hour. Not including RERAs in the calculation remains an ‘acceptable’ scoring criterion as per the AASM, which aligns with the insurance policies set by the Centers for Medicare and Medical Services [40]. This discrepancy may result in symptomatic patients being overlooked for treatment when AHI is used alone. Studies also show that the RERA index is significantly correlated with overall AHI, underscoring its importance in symptomatic individuals [41].

To recognize the clinical impact of RERAs in sleep disordered breathing, Guilleminault introduced the term Upper Airway Resistance Syndrome (UARS) [42], which describes the RERA predominant OSA phenotype. Individuals with UARS are more likely to experience symptoms such as difficulty initiating or maintaining sleep, postural hypotension, headaches, gastroesophageal reflux, irritable bowel syndrome, anxiety, and alpha-delta sleep (Table 2) [43,44,45]. This emphasizes the importance of highlighting RDI in sleep studies to ensure a comprehensive evaluation and effective management of RERA-predominant OSA or UARS.

Among the pediatric population, the characteristic esophageal pressure negativity in UARS is noted to influence hemodynamics, through which it can cause a diastolic leftward shift of an interventricular septum and thereby, ventricular collapse [46]. Untreated UARS can induce a small increase in end tidal carbon dioxide which can lead to increased sympathetic nervous system activity and thereby worse cardiometabolic consequences [47]. These patients have been mostly treated using PAP therapy, which is known to have clinical benefit, despite the limited insurance coverage [48]. A study of postmenopausal women with chronic insomnia and UARS showed improvement in daytime fatigue levels after the nasal CPAP treatment [49], highlighting the importance of its management, if present with other sleep disorders.

### 3.5. Phenotyping by Oxygenation Metrics

Measuring OSA severity by hypoxic burden rather than AHI may provide a more accurate assessment of risk. Hypoxic burden can be quantified using metrics such as the oxygen desaturation index (ODI), the area under the desaturation curve, and the proportion of sleep time spent with an oxygen saturation below 90%. While sustained hypoxemia (oxygen saturation below 88% for more than 5 min) can occur in conditions such as rapid altitude ascent, obesity hypoventilation syndrome or chronic lung disease, but this is distinct from the intermittent hypoxemia seen in OSA, primarily because of the cyclical re-oxygenation patterns and increased sympathetic nervous system activity associated with it. These cycles resemble ischemia-reperfusion injury and contribute to elevated levels of reactive oxygen species and oxidative stress [50]. Measuring the hypoxic burden predicts cardiovascular mortality and all-cause mortality, and this hypothesis was tested in the Osteoporotic Fractures in Men Study and the Sleep Heart Health Study. The total area under the ventilation curve, using eupneic ventilation as the baseline is a reliable indicator to quantify the total ventilatory deficit caused by obstruction in the airway. Analyses show that the hypoxemia burden is the strongest predictor of major adverse cardiovascular events, surpassing AHI (Table 2) [51]. 

The strongest OSA-related predictor of cardiovascular events or all-cause mortality was total sleep time spent with oxygen saturation below 90% (T < 90), which raised the risk of a cardiovascular event or death by 50%. This magnitude of risk is not reflected when using AHI alone. For instance, spending 9 min below 90% oxygen saturation compared to none is linked to a 58% increase in mortality risk [52]. Patients with higher levels of nocturnal hypoxemia also appear to receive greater long-term cardiovascular benefit from PAP therapy, further supporting oxygen metrics as an important phenotype [53]. Sleep oxygen saturation levels of ≤ 60% have also been identified as posing a higher risk of cardiovascular disease and an increased propensity for ventricular arrhythmias [54]. An observational cohort study based on a machine learning model has also shown nocturnal hypoxemia to be the main risk factor for mortality after adjustment of confounding factors [55]. Classification of hypopneas by degree of oxygen desaturation remains debated, especially regarding the clinical significance of 3% versus 4% drops during events. Some analyses suggest that only hypopneas causing at least a 4% desaturation are associated with cardiovascular disease [56,57]. However, the hypopnea predominant-OSA phenotype with hypopnea described by the 3% desaturation or arousal rule was associated with coronary artery disease and heart failure in a retrospective study [26].

### 3.6. Phenotyping by Arousal and Sleep Fragmentation

Sleep fragmentation due to arousals has independent clinical significance. The arousal index correlates with sympathetic overactivity [58,59] and is associated with carotid and coronary artery plaques [60], high coronary artery calcification burden [61], and hypertension [62]. Additionally, respiratory events with arousals, compared to ones without, had a higher influence in increasing blood pressure, highlighting the importance of evaluating RERAs and their role in OSA phenotyping. Additionally, a long duration of wake time after sleep onset and irregular sleep duration is closely associated with adverse cardiovascular outcomes [63,64]. Periodic limb movements during sleep are noted to be an independent risk factor for incident cardiovascular disease and mortality after adjusting for appropriate confounders, especially in the patients with no or mild to moderate OSA [65]. The proposed mechanism is cyclical episodes of oxygen desaturation in the brain due to repetitive changes in the cerebral hemodynamics associated with it [66].

## 4. Discussion

The findings from this review highlight the complexity of OSA and reveal that relying on AHI alone fails to capture the full spectrum of disease severity. Polysomnography offers the opportunity to collect a myriad of physiological parameters which must be considered along with AHI to subclassify OSA in these phenotypes which would better reflect the type and severity of OSA. When only AHI is considered, the reflexive prescription of positive airway pressure therapy for elevated AHI may lead to mistreatment of sleep disordered breathing. This can further lead to PAP compliance issues which can then propagate the various misconceptions about PAP therapy and overall sleep apnea care. Conversely, individuals with supine- or REM-predominant OSA may go undertreated if their total AHI is below the threshold, potentially exposing them to long-term cardiovascular risks.

This review emphasizes the Q2 analysis of OSA (Table 1) and advocates a detailed breakdown of polysomnographic findings that goes beyond traditional measures such as AHI and oxygen desaturation. We suggest stratifying AHI based on respiratory event type and duration, sleep stage, and body-position. Incorporating arousal index into the reporting will open avenues for the treatment of hyperarousal using hypnotic agents, if related to sleep disordered breathing. A comprehensive approach to phenotyping will pave the way for future research, allowing for a better understanding of causal links between phenotypes and cardio-neuro-metabolic outcomes (Table 3). These models could be used in machine learning platforms which may help with stratification of adverse cardiovascular events and mortality risk for each phenotype. One such observational study involving machine learning helped identify certain clusters of OSA based on the PSG data and history of cardio-neuro-metabolic diseases which may help with automated assistance in the diagnosis, risk assessment, and treatment of OSA [67]. Currently, there remains a lack of prospective data, randomized controlled trials, and epidemiological studies assessing the long-term consequences of these phenotypes.

Implementing Q2 analysis in clinical practice comes with several challenges. For example, current Centers for Medicare Services require a 4% oxygen desaturation threshold to define hypopneas potentially overlooking cases involving 3% desaturation events and leading to underdiagnosis. Recognizing RERAs and using RDI may assist in identification of phenotype with RDI predominant sleep apnea yet these individuals may have overall AHI values below 5 events per hour, which can impact insurance reimbursement for therapy. Another challenge lies in the epidemiologic screening of sleep disordered breathing, especially in high-risk populations. Despite the prevalence of OSA being as high as 40–80% in patients with hypertension, heart failure, stroke, coronary artery disease, atrial fibrillation, and pulmonary hypertension [68], there remain system-based deficiencies in adequate screening of these patients due to multiple institutional and individual barriers. Innovative approaches such as the integrated mobile sleep medicine model [69], can improve identification and screening of patients at high risk of obstructive sleep apnea and sleep disordered breathing using different types of sleep studies, with appropriate testing and follow up. This initiative can help screen more patients with a high risk of sleep disordered breathing and help add to the different phenotypes of sleep apnea in these patient groups. Moving toward precision sleep medicine in OSA has the potential to optimize patient outcomes and resource allocation.

Understanding of phenotypes will undoubtedly enhance the practice of sleep medicine and help personalize therapies. Lessons from other conditions, such as asthma, where cluster analysis and identification of endotypes have informed the use of biologics for symptomatic patients with eosinophilia [70]. Incorporation of polysomnographic metrics beyond AHI is required, which can greatly assist in prognostication of OSA and help explore alternative therapeutic options. There remains an unmet need to document the Q2 data on OSA phenotypes with future prospective epidemiological studies.

## 5. Conclusions

OSA is a heterogeneous disorder that cannot be adequately captured by AHI alone. Incorporating phenotypic subclassification based on Q2 analysis—including respiratory event predominance, duration, REM/supine dependence, neurophysiological traits, hypoxic burden, and arousal indices—enhances diagnostic accuracy, refines prognostication, and enables personalized therapy. Embracing phenotyping in both clinical practice and research is essential to advance precision medicine in sleep apnea. Previous investigations have demonstrated results linking higher AHI to increased cardiovascular mortality, but there remain challenges for the integration of phenotypic parameters into clinical guidelines, developing composite indices, and designing phenotype-targeted therapeutic trials.

## Figures and Tables

**Table 1:** Parameters for subclassification of respiratory events, with quality and quantity (Q2) measures (RDI, respiratory disturbance index; OAI, obstructive apnea index; OHI, obstructive hypopnea index; RERA, respiratory event related arousal; AHI, apnea hypopnea index; REM, rapid eye movement; NREM, non-rapid eye movement; PLMD; periodic limb movement disorder; RLS, restless leg syndrome).

Parameter	Quality	Quantity
Respiratory events	Predominance of event in RDI - Obstructive apnea (OAI > 50%) - Obstructive hypopnea (OHI > 50%) - RERA (RERA > 50%) Combined events (33–50%) in RDI without isolated predominance - Obstructive apnea + obstructive hypopnea - Obstructive apnea + RERA - Obstructive hypopnea + RERA Predominance of event in AHI - Obstructive apnea (OAI > 50%) - Obstructive hypopnea (OHI > 50%)	Number of obstructive respiratory events (AHI/RDI) (events/hour) - Mild (5–15) - Moderate (15–30) - Severe (>30) Supine predominant OSA (supine AHI/non supine AHI ratio > 2, total AHI > 5) - Mild (5–15) - Moderate (15–30) - Severe (>30) REM predominant OSA (REM AHI/NREM AHI ratio > 2, total AHI > 5) - Mild (5–15) - Moderate (15–30) - Severe (>30) Duration of event - 10–20 s - 20–30 s - >30 s
Oxygen	Hypoxia burden (Area under the curve)	Oxygen desaturation index - Mild (5–15) - Moderate (15–30) - Severe (>30) Desaturation nadir
Arousal	Respiratory Event Related Arousal (RERA) Limb Movement Related Arousal (PLMD, RLS)	Total arousal index - Mild (5–15) - Moderate (15–30) - Severe (>30) Mean duration of arousal Wake after sleep onset (WASO)

**Table 2:** Summary of phenotypes, clinical consequences, and therapeutic implications. (CPAP, continuous positive airway pressure; CAD, coronary artery disease; CHF, congestive heart failure; GERD, gastroesophageal reflux disease; PAP, positive airway pressure; OSA, obstructive sleep apnea; CV, cardiovascular).

Phenotype	Clinical Consequences	Therapeutic Implications
Apnea-predominant	Static obstruction, higher oxygen desaturation index, high level of excessive daytime sleepiness, hypertension, diabetes	Standard CPAP, oral appliances
Hypopnea-predominant	Dynamic obstruction, older individuals, more prevalent in females, higher risk of CAD, CHF, dyslipidemia risk	Surgical interventions (e.g., maxillomandibular advancement)
RERA-predominant/UARS	Daytime sleepiness, neurocognitive symptoms, insomnia, headaches, GERD	PAP therapy (may be underrecognized due to AHI reliance), arousal suppressing medications
Long-duration events	Long-duration apneas have louder snoring, more morning tiredness, and higher risk of hypertension	Aggressive treatment targeting residual events
Short-duration events	More commonly seen with severe OSA, 15–59% increase in risk of mortality with short-duration events in moderate OSA, shorter hypopneas noted in severe OSA compared to milder severity groups	PAP therapy
REM-predominant	Hypertension, recurrent CV events, more prevalent in women	CPAP even if overall AHI < 5
Supine-predominant	Marked positional dependence	Positional therapy, auto-PAP
High hypoxic burden	Strongest predictor of CV mortality	Greatest benefit from CPAP
High arousal burden	Sympathetic activation, atherosclerosis	Target arousal threshold, optimize sleep continuity

**Table 3:** Current evidence and research gaps in the qualitative and quantitative (Q2) analysis of phenotypes of OSA. (AHI, apnea hypopnea index; RERA, respiratory event related arousal; OSA, obstructive sleep apnea; REM, rapid eye movement).

OSA Clinical Phenotypes	Current Research	Research Gaps
Respiratory event type	Retrospective studies split AHI into apnea, hypopnea, and RERA-predominant OSA and associated them with comorbidities.	Lack of prospective data, no randomized control or epidemiological trials No research separating compliant and non-compliant groups
Respiratory event duration	Shorter event duration associated with increased mortality from retrospective cohort study, but greater hemodynamic fluctuations in cerebral blood flow are noted in longer events.	Lack of prospective data, no randomized control or epidemiological trials No research separating compliant and non-compliant groups No research about the change in heart rate/arrythmia associated with the respiratory event and its association with cardiovascular outcomes
Based on sleep stage and body position	REM predominant OSA more common in women and is associated with prevalent hypertension as per retrospective cohort study. Variations in airway collapsibility in supine-predominant OSA better treated with auto PAP	Lack of prospective data, no randomized control or epidemiological trials No research separating compliant and non-compliant groups Lack of data of predominance of OSA in stages other than R (such as stage N1, N2, or N3) Lack of data in lateral or prone position-predominant OSA
Neurophysiological traits	UARS establishes RERA-predominant phenotype, these patients have significantly more symptoms including insomnia and neurocognitive symptoms	Lack of prospective data, no randomized control or epidemiological trials No research separating compliant and non-compliant groups No data about duration of RERA and adverse cardiovascular events
Oxygenation metrics	Strongest OSA-related predictor of major adverse cardiovascular events and mortality. Average oxygen desaturation depth increases as a function of AHI	Lack of prospective data, no randomized control or epidemiological trials No research separating compliant and non-compliant groups No differentiation in patients on sleep-time oxygen therapy treating hypoxemia versus obstructive sleep apnea
Arousal and sleep fragmentation	High arousal burden linked to sympathetic activation and atherosclerosis	Lack of prospective data, no randomized control or epidemiological trials No research separating compliant and non-compliant groups

## Data Availability

No new data were created or analyzed in this study. Data sharing is not applicable to this article.

## References

[1] Maspero C., Giannini L., Galbiati G., Rosso G., Farronato G. (2015). Obstructive sleep apnea syndrome: A literature review. Minerva Stomatol..

[2] Benjafield A.V., Ayas N.T., Eastwood P.R., Heinzer R., Ip M.S.M., Morrell M.J., Nunez C.M., Patel S.R., Penzel T., Pepin J.L. (2019). Estimation of the global prevalence and burden of obstructive sleep apnoea: A literature-based analysis. Lancet Respir. Med..

[3] Kapur V.K., Auckley D.H., Chowdhuri S., Kuhlmann D.C., Mehra R., Ramar K., Harrod C.G. (2017). Clinical Practice Guideline for Diagnostic Testing for Adult Obstructive Sleep Apnea: An American Academy of Sleep Medicine Clinical Practice Guideline. J. Clin. Sleep Med..

[4] Yacoub M., Youssef I., Salifu M.O., McFarlane S.I. (2017). Cardiovascular Disease Risk in Obstructive Sleep apnea: An Update. J. Sleep Disord. Ther..

[5] Young T., Finn L., Peppard P.E., Szklo-Coxe M., Austin D., Nieto F.J., Stubbs R., Hla K.M. (2008). Sleep disordered breathing and mortality: Eighteen-year follow-up of the Wisconsin sleep cohort. Sleep.

[6] Arzt M., Young T., Finn L., Skatrud J.B., Bradley T.D. (2005). Association of sleep-disordered breathing and the occurrence of stroke. Am. J. Respir. Crit. Care Med..

[7] Cananzi S.G., White L.A., Barzegar M., Boyer C.J., Chernyshev O.Y., Yun J.W., Kelley R.E., Almendros I., Minagar A., Farre R. (2020). Obstructive sleep apnea intensifies stroke severity following middle cerebral artery occlusion. Sleep Med..

[8] Mazzotti D.R., Waitman L.R., Miller J., Sundar K.M., Stewart N.H., Gozal D., Song X., Greater Plains C. (2024). Positive Airway Pressure, Mortality, and Cardiovascular Risk in Older Adults with Sleep Apnea. JAMA Netw. Open.

[9] Wickwire E.M., Bailey M.D., Somers V.K., Srivastava M.C., Scharf S.M., Johnson A.M., Albrecht J.S. (2021). CPAP adherence is associated with reduced risk for stroke among older adult Medicare beneficiaries with obstructive sleep apnea. J. Clin. Sleep Med..

[10] Benjafield A.V., Pepin J.L., Cistulli P.A., Wimms A., Lavergne F., Sert Kuniyoshi F.H., Munson S.H., Schuler B., Reddy Badikol S., Wolfe K.C. (2025). Positive airway pressure therapy and all-cause and cardiovascular mortality in people with obstructive sleep apnoea: A systematic review and meta-analysis of randomised controlled trials and confounder-adjusted, non-randomised controlled studies. Lancet Respir. Med..

[11] Peker Y., Glantz H., Eulenburg C., Wegscheider K., Herlitz J., Thunstrom E. (2016). Effect of Positive Airway Pressure on Cardiovascular Outcomes in Coronary Artery Disease Patients with Nonsleepy Obstructive Sleep Apnea. The RICCADSA Randomized Controlled Trial. Am. J. Respir. Crit. Care Med..

[12] McEvoy R.D., Antic N.A., Heeley E., Luo Y., Ou Q., Zhang X., Mediano O., Chen R., Drager L.F., Liu Z. (2016). CPAP for Prevention of Cardiovascular Events in Obstructive Sleep Apnea. N. Engl. J. Med..

[13] Sanchez-de-la-Torre M., Sanchez-de-la-Torre A., Bertran S., Abad J., Duran-Cantolla J., Cabriada V., Mediano O., Masdeu M.J., Alonso M.L., Masa J.F. (2020). Effect of obstructive sleep apnoea and its treatment with continuous positive airway pressure on the prevalence of cardiovascular events in patients with acute coronary syndrome (ISAACC study): A randomised controlled trial. Lancet Respir. Med..

[14] Randerath W., Bassetti C.L., Bonsignore M.R., Farre R., Ferini-Strambi L., Grote L., Hedner J., Kohler M., Martinez-Garcia M.A., Mihaicuta S. (2018). Challenges and perspectives in obstructive sleep apnoea: Report by an ad hoc working group of the Sleep Disordered Breathing Group of the European Respiratory Society and the European Sleep Research Society. Eur. Respir. J..

[15] Zinchuk A., Yaggi H.K. (2020). Phenotypic Subtypes of OSA: A Challenge and Opportunity for Precision Medicine. Chest.

[16] Malhotra A., Ayappa I., Ayas N., Collop N., Kirsch D., McArdle N., Mehra R., Pack A.I., Punjabi N., White D.P. (2021). Metrics of sleep apnea severity: Beyond the apnea-hypopnea index. Sleep.

[17] Owens R.L., Edwards B.A., Eckert D.J., Jordan A.S., Sands S.A., Malhotra A., White D.P., Loring S.H., Butler J.P., Wellman A. (2015). An Integrative Model of Physiological Traits Can be Used to Predict Obstructive Sleep Apnea and Response to Non Positive Airway Pressure Therapy. Sleep.

[18] Wellman A., Malhotra A., Jordan A.S., Stevenson K.E., Gautam S., White D.P. (2008). Effect of oxygen in obstructive sleep apnea: Role of loop gain. Respir. Physiol. Neurobiol..

[19] Edwards B.A., Sands S.A., Eckert D.J., White D.P., Butler J.P., Owens R.L., Malhotra A., Wellman A. (2012). Acetazolamide improves loop gain but not the other physiological traits causing obstructive sleep apnoea. J. Physiol..

[20] Strollo P.J., Soose R.J., Maurer J.T., de Vries N., Cornelius J., Froymovich O., Hanson R.D., Padhya T.A., Steward D.L., Gillespie M.B. (2014). Upper-airway stimulation for obstructive sleep apnea. N. Engl. J. Med..

[21] Tolbert T.M., Schmickl C.N., Gell L.K., Keenan B.T., Mazzotti D.R., Op de Beeck S., Osman A.M., Pena-Orbea C., Sands S.A., Sofer T. (2025). Research Priorities for Translating Endophenotyping of Adult Obstructive Sleep Apnea to the Clinic: An Official American Thoracic Society Research Statement. Am. J. Respir. Crit. Care Med..

[22] Shahar E. (2014). Apnea-hypopnea index: Time to wake up. Nat. Sci. Sleep.

[23] Bosi M., De Vito A., Gobbi R., Poletti V., Vicini C. (2017). The importance of obstructive sleep apnoea and hypopnea pathophysiology for customized therapy. Eur. Arch. Otorhinolaryngol..

[24] Montserrat J.M., Farre R., Navajas D. (2001). New technologies to detect static and dynamic upper airway obstruction during sleep. Sleep. Breath..

[25] Mathew R., Castriotta R.J. (2014). High hypopnea/apnea ratio (HAR) in extreme obesity. J. Clin. Sleep Med..

[26] Park S., Shin B., Lee J.H., Lee S.J., Lee M.K., Lee W.Y., Yong S.J., Kim S.H. (2020). Polysomnographic phenotype as a risk factor for cardiovascular diseases in patients with obstructive sleep apnea syndrome: A retrospective cohort study. J. Thorac. Dis..

[27] Spector A.R., Loriaux D., Farjat A.E. (2019). The Clinical Significance of Apneas Versus Hypopneas: Is There Really a Difference?. Cureus.

[28] Ho J.T.F., Zhou N., de Lange J. (2022). Obstructive Sleep Apnea Resolution in Hypopnea-Predominant versus Apnea-Predominant Patients after Maxillomandibular Advancement. J. Clin. Med..

[29] Alex R., Manchikatla S., Machiraju K., Altuwaijri E., Watenpaugh D.E., Zhang R., Behbehani K. Effect of apnea duration on apnea induced variations in cerebral blood flow velocity and arterial blood pressure. Proceedings of the Annual International Conference of the IEEE Engineering in Medicine and Biology Society.

[30] Butler M.P., Emch J.T., Rueschman M., Sands S.A., Shea S.A., Wellman A., Redline S. (2019). Apnea-Hypopnea Event Duration Predicts Mortality in Men and Women in the Sleep Heart Health Study. Am. J. Respir. Crit. Care Med..

[31] Oksenberg A., Leppanen T. (2023). Duration of respiratory events in obstructive sleep apnea: In search of paradoxical results. Sleep Med. Rev..

[32] Sarac S., Afsar G.C. (2020). Effect of mean apnea-hypopnea duration in patients with obstructive sleep apnea on clinical and polysomnography parameter. Sleep Breath..

[33] Bonsignore M.R., Mazzuca E., Baiamonte P., Bouckaert B., Verbeke W., Pevernagie D.A. (2024). REM sleep obstructive sleep apnoea. Eur. Respir. Rev..

[34] Douglas N.J. (1985). Control of ventilation during sleep. Clin. Chest Med..

[35] Mokhlesi B., Finn L.A., Hagen E.W., Young T., Hla K.M., Van Cauter E., Peppard P.E. (2014). Obstructive sleep apnea during REM sleep and hypertension. results of the Wisconsin Sleep Cohort. Am. J. Respir. Crit. Care Med..

[36] Aurora R.N., Crainiceanu C., Gottlieb D.J., Kim J.S., Punjabi N.M. (2018). Obstructive Sleep Apnea during REM Sleep and Cardiovascular Disease. Am. J. Respir. Crit. Care Med..

[37] Joosten S.A., Edwards B.A., Wellman A., Turton A., Skuza E.M., Berger P.J., Hamilton G.S. (2015). The Effect of Body Position on Physiological Factors that Contribute to Obstructive Sleep Apnea. Sleep.

[38] Series F., Marc I. (2001). Importance of sleep stage- and body position-dependence of sleep apnoea in determining benefits to auto-CPAP therapy. Eur. Respir. J..

[39] Koch H., Schneider L.D., Finn L.A., Leary E.B., Peppard P.E., Hagen E., Sorensen H.B.D., Jennum P., Mignot E. (2017). Breathing Disturbances Without Hypoxia Are Associated with Objective Sleepiness in Sleep Apnea. Sleep.

[40] Malhotra R.K., Kirsch D.B., Kristo D.A., Olson E.J., Aurora R.N., Carden K.A., Chervin R.D., Martin J.L., Ramar K., Rosen C.L. (2018). Polysomnography for Obstructive Sleep Apnea Should Include Arousal-Based Scoring: An American Academy of Sleep Medicine Position Statement. J. Clin. Sleep Med..

[41] Ghandeharioun H., Rezaeitalab F., Lotfi R. (2016). Analysis of respiratory events in obstructive sleep apnea syndrome: Inter-relations and association to simple nocturnal features. Rev. Port. Pneumol..

[42] Guilleminault C., Stoohs R., Clerk A., Cetel M., Maistros P. (1993). A cause of excessive daytime sleepiness. The upper airway resistance syndrome. Chest.

[43] Gold A.R., Dipalo F., Gold M.S., O’Hearn D. (2003). The symptoms and signs of upper airway resistance syndrome: A link to the functional somatic syndromes. Chest.

[44] Guilleminault C., Stoohs R., Kim Y.D., Chervin R., Black J., Clerk A. (1995). Upper airway sleep-disordered breathing in women. Ann. Intern. Med..

[45] Guilleminault C., Black J.E., Palombini L., Ohayon M. (2000). A clinical investigation of obstructive sleep apnea syndrome (OSAS) and upper airway resistance syndrome (UARS) patients. Sleep Med..

[46] Shiomi T., Guilleminault C., Stoohs R., Schnittger I. (1993). Obstructed breathing in children during sleep monitored by echocardiography. Acta Paediatr..

[47] Pepin J.L., Guillot M., Tamisier R., Levy P. (2012). The upper airway resistance syndrome. Respiration.

[48] de Godoy L.B., Palombini L.O., Guilleminault C., Poyares D., Tufik S., Togeiro S.M. (2015). Treatment of upper airway resistance syndrome in adults: Where do we stand?. Sleep Sci..

[49] Guilleminault C., Palombini L., Poyares D., Chowdhuri S. (2002). Chronic insomnia, premenopausal women and sleep disordered breathing: Part 2. Comparison of nondrug treatment trials in normal breathing and UARS post menopausal women complaining of chronic insomnia. J. Psychosom. Res..

[50] Dewan N.A., Nieto F.J., Somers V.K. (2015). Intermittent hypoxemia and OSA: Implications for comorbidities. Chest.

[51] Azarbarzin A., Sands S.A., Stone K.L., Taranto-Montemurro L., Messineo L., Terrill P.I., Ancoli-Israel S., Ensrud K., Purcell S., White D.P. (2019). The hypoxic burden of sleep apnoea predicts cardiovascular disease-related mortality: The Osteoporotic Fractures in Men Study and the Sleep Heart Health Study. Eur. Heart J..

[52] Kendzerska T., Gershon A.S., Hawker G., Leung R.S., Tomlinson G. (2014). Obstructive sleep apnea and risk of cardiovascular events and all-cause mortality: A decade-long historical cohort study. PLoS Med..

[53] Pinilla L., Esmaeili N., Labarca G., Martinez-Garcia M.A., Torres G., Gracia-Lavedan E., Minguez O., Martinez D., Abad J., Masdeu M.J. (2023). Hypoxic burden to guide CPAP treatment allocation in patients with obstructive sleep apnoea: A post hoc study of the ISAACC trial. Eur. Respir. J..

[54] Zwillich C., Devlin T., White D., Douglas N., Weil J., Martin R. (1982). Bradycardia during sleep apnea. Characteristics and mechanism. J. Clin. Investig..

[55] Tondo P., Scioscia G., Bailly S., Sabato R., Campanino T., Soccio P., Foschino Barbaro M.P., Gallo C., Pepin J.L., Lacedonia D. (2025). Exploring phenotypes to improve long-term mortality risk stratification in obstructive sleep apnea through a machine learning approach: An observational cohort study. Eur. J. Intern. Med..

[56] Parekh A. (2024). Hypoxic burden—Definitions, pathophysiological concepts, methods of evaluation, and clinical relevance. Curr. Opin. Pulm. Med..

[57] Punjabi N.M., Newman A.B., Young T.B., Resnick H.E., Sanders M.H. (2008). Sleep-disordered breathing and cardiovascular disease: An outcome-based definition of hypopneas. Am. J. Respir. Crit. Care Med..

[58] Kim J.B., Seo B.S., Kim J.H. (2019). Effect of arousal on sympathetic overactivity in patients with obstructive sleep apnea. Sleep Med..

[59] Taylor K.S., Murai H., Millar P.J., Haruki N., Kimmerly D.S., Morris B.L., Tomlinson G., Bradley T.D., Floras J.S. (2016). Arousal From Sleep and Sympathetic Excitation During Wakefulness. Hypertension.

[60] Lu M., Yu W., Wang Z., Huang Z. (2022). Association between Arousals during Sleep and Subclinical Coronary Atherosclerosis in Patients with Obstructive Sleep Apnea. Brain Sci..

[61] Suzuki M., Shimamoto K., Sekiguchi H., Harada T., Satoya N., Inoue Y., Yamaguchi K., Kawana M. (2019). Arousal index as a marker of carotid artery atherosclerosis in patients with obstructive sleep apnea syndrome. Sleep Breath..

[62] Ren R., Zhang Y., Yang L., Somers V.K., Covassin N., Tang X. (2022). Association Between Arousals During Sleep and Hypertension Among Patients with Obstructive Sleep Apnea. J. Am. Heart Assoc..

[63] Yan B., Yang J., Zhao B., Fan Y., Wang W., Ma X. (2021). Objective Sleep Efficiency Predicts Cardiovascular Disease in a Community Population: The Sleep Heart Health Study. J. Am. Heart Assoc..

[64] Huang T., Mariani S., Redline S. (2020). Sleep Irregularity and Risk of Cardiovascular Events: The Multi-Ethnic Study of Atherosclerosis. J. Am. Coll. Cardiol..

[65] Zinchuk A., Srivali N., Qin L., Jeon S., Ibrahim A., Sands S.A., Koo B., Yaggi H.K. (2024). Association of Periodic Limb Movements and Obstructive Sleep Apnea with Risk of Cardiovascular Disease and Mortality. J. Am. Heart Assoc..

[66] Boulos M.I., Murray B.J., Muir R.T., Gao F., Szilagyi G.M., Huroy M., Kiss A., Walters A.S., Black S.E., Lim A.S. (2017). Periodic Limb Movements and White Matter Hyperintensities in First-Ever Minor Stroke or High-Risk Transient Ischemic Attack. Sleep.

[67] Ma E.-Y., Kim J.-W., Lee Y., Cho S.-W., Kim H., Kim J.K. (2021). Combined unsupervised-supervised machine learning for phenotyping complex diseases with its application to obstructive sleep apnea. Sci. Rep..

[68] Javaheri S., Barbe F., Campos-Rodriguez F., Dempsey J.A., Khayat R., Javaheri S., Malhotra A., Martinez-Garcia M.A., Mehra R., Pack A.I. (2017). Sleep Apnea: Types, Mechanisms, and Clinical Cardiovascular Consequences. J. Am. Coll. Cardiol..

[69] Boulos M.I., Chi L., Chernyshev O.Y. (2022). The mobile sleep medicine model in neurologic practice: Rationale and application. Front. Neurol..

[70] Moore W.C., Meyers D.A., Wenzel S.E., Teague W.G., Li H., Li X., D’Agostino R., Castro M., Curran-Everett D., Fitzpatrick A.M. (2010). Identification of asthma phenotypes using cluster analysis in the Severe Asthma Research Program. Am. J. Respir. Crit. Care Med..

